# Supplementary material for: A Synthetic Cell-Penetrating Heparin-Binding Peptide Derived from BMP4 with Anti-Inflammatory and Chondrogenic Functions for the Treatment of Arthritis
Source: Int J Mol Sci. 2020 Jun 15;21(12):4251. doi: 10.3390/ijms21124251 (PMC7352680; doi:10.3390/ijms21124251)
Supplement: Supplementary file 1 [file ijms-21-04251-s001.zip › ijms-831941 suppl for final 2/Supple_Figures.pdf]

A

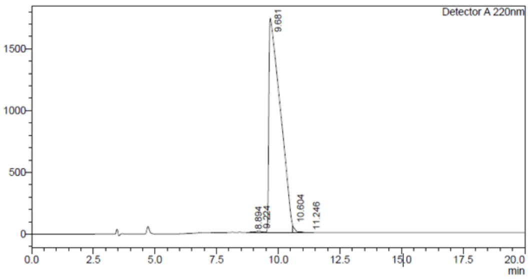

<Peak Table>

Detector A 220nm

Peak#	Ret. Time	Area	Height	Area%	Height%
1	8.894	28720	4066	0.051	0.226
2	9.224	195408	13096	0.344	0.726
3	9.681	56214614	1733776	98.982	96.174
4	10.604	345852	50796	0.609	2.818
5	11.246	8189	1016	0.014	0.056
Total		56792783	1802750	100.000	100.000

B

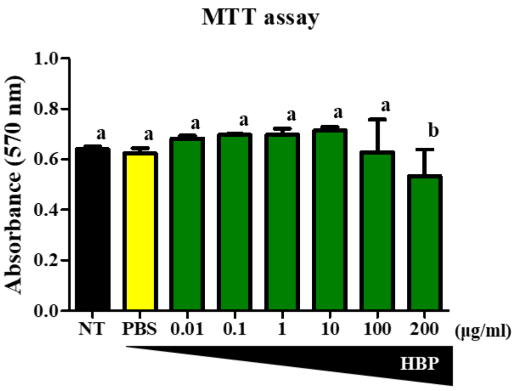

Supplementary Figure S1. (**A**) The high pressure liquid chromatogram (HPLC) of HBP. The retention time of HBP was 9.681min and main peak area of HBP was 98.9%. (**B**) The cytotoxicity of HBP in RAW 264.7 cells. Cells were incubated with various concentrations of HBP for 14 hr. The number of viable cells remaining in the wells was assessed using the MTT assay and compared with non-treated cells (NT). The data are expressed as the mean ± S.D. from three independent experiments in each group. Different alphabets (a and b) indicate significant differences among experimental groups ($p < 0.05$).

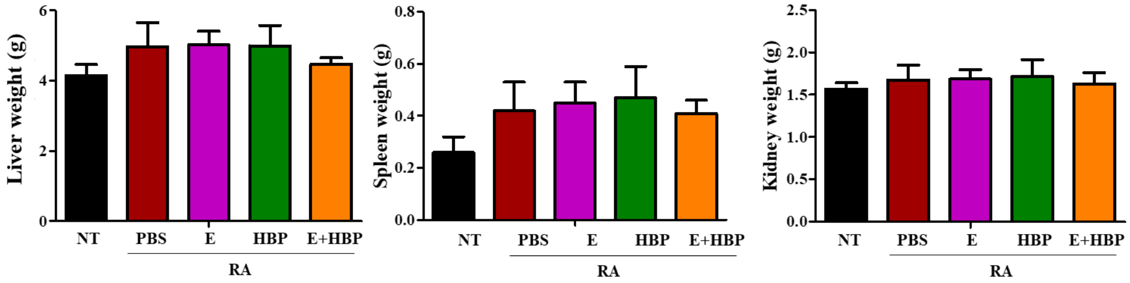

Supplementary Figure S2. The organ weights in mouse. Data are expressed as Mean ± S.D. When the necropsy was performed, the liver, spleen, and kidney were separated from the body and measured the weights. The selected results were statistically analyzed by one-way ANOVA methods ($n = 10$ per group). There was no significant change between each group.
