# Supplementary material for: A Synthetic Cell-Penetrating Heparin-Binding Peptide Derived from BMP4 with Anti-Inflammatory and Chondrogenic Functions for the Treatment of Arthritis
Source: Int J Mol Sci. 2020 Jun 15;21(12):4251. doi: 10.3390/ijms21124251 (PMC7352680; doi:10.3390/ijms21124251)
Supplement: Supplementary file 1 [file ijms-21-04251-s001.zip › ijms-831941 suppl for final 2/Suppl_Table.pdf]

Supplementary Table S1. Sequences of primers used for RT-qPCR.

Gene	Primer Sequence (5′ - 3′)
AGG	F: CAACAACAATGCCCAAGACTAC
	R: AGTTCTCAAATTGCAAGGAGTG
TNFα	F: CACAGTGAAGTGCTGGCAAC
	R: AGGAAGGCCTAAGGTCCACT
COLII	F: CTATCTGGACGAAGCAGCTGGCA
	R: ATGGGTGCAATGTCAATGATGG
GAPDH	F: ACATCGCTCAGACACCATG
	R: TGTAGTTGAGGTCAATGAAGGG

AGG (Aggrecan), TNFα (Tumor necrosis factor alpha), COLII (Type-II Collagen), GAPDH (glyceraldehyde-3-phosphate dehydrogenase)
